# O003: The misuse of clinical gloves: risk of cross-infection and factors influencing the decision of healthcare workers to wear gloves

**DOI:** 10.1186/2047-2994-2-S1-O3

**Published:** 2013-06-20

**Authors:** J Wilson, S Lynam, J Singleton, H Loveday

**Affiliations:** 1 Institute of Practice, Interdisciplinary Research & Enterprise, UK; 2 Psychology, Social Care & Human Sciences, University of West London, UK; 3 Infection Control Directorate, Imperial College Healthcare NHS Trust, UK; 4 Richard Wells Research Centre, University of West London, London, UK

## Introduction

Clinical gloves are routinely used in the delivery of patient care but unless integrated with the ‘5 moments of hand hygiene’ have the potential to increase the risk of HCAI transmission.

## Objectives

To examine glove use in an acute care setting, the extent to which they are associated with a risk of cross contamination, and factors that influence healthcare workers (HCW) decision to wear them.

## Methods

Observation of the use of clinical gloves was conducted in 6 wards by two trained observers. Independent observations were compared for inter-rater reliability. Glove use was considered appropriate if the episode involved potential contact with blood/body fluid (BBF). Risk of cross contamination was defined as violation of one or more of the ‘moments of hand hygiene’ during the glove-use episode. Semi-structured interviews were conducted with a purposive sample of 25 HCW from audited wards to explore attitudes towards the use of gloves.

## Results

164 glove use episodes were observed over 13 hours. Glove use was appropriate in 58% (95/164) of episodes, but gloves were commonly used for procedures with minimal risk of exposure to BBF. In 39% of glove-use episodes there was a risk of cross contamination, this was significantly more likely to occur where gloves were used inappropriately (58.4% vs 28.4%; Chi^2^ p <0.01). In 24% (39) episodes more than 5 objects were touched by a gloved hand before the procedure was performed. In one third of episodes, hand hygiene was not performed after glove removal. The key themes from qualitative interviews with HCW indicated that the decision to wear gloves was influenced by multidimensional socialisation and emotion. Key emotions were disgust and fear, but assumptions about patients and their preferences regards glove use, confusion about when to wear them and peer pressure, were also important influences.

## Conclusion

Glove use in acute clinical settings is associated with a significant risk of cross contamination and needs to be more explicitly integrated into hand hygiene policy. An understanding of drivers of glove use behaviour is required to design interventions to reduce their misuse and overuse.

## Disclosure of interest

None declared.

